# ZnCdO:Eu Epitaxially Grown Alloys for Self-Powered Ultrafast Broadband Photodetection

**DOI:** 10.1021/acsami.6c01143

**Published:** 2026-03-25

**Authors:** Igor Perlikowski, Eunika Zielony, Aleksandra Wierzbicka, Adrian Kaim, Anastasiia Lysak, Rafał Jakieła, Yaroslav Zhydachevskyy, Ewa Przeździecka

**Affiliations:** † Department of Experimental Physics, 49567 Wrocław University of Science and Technology, Wybrzeże Wyspiańskiego 27, Wrocław 50-370, Poland; ‡ 86906 Institute of Physics, Polish Academy of Sciences, Al. Lotników 32/46, Warsaw 02-668, Poland

**Keywords:** rare-earth-doped films, molecular beam epitaxy, ZnCdO alloy, ultrafast photodetectors, pyro-phototronic effect

## Abstract

Photodetectors (PDs) are essential in imaging, communication, and sensing technologies. However, their reliance on external power makes them energy-consuming. This creates a strong need for self-powered PDs as a sustainable alternative. Zinc oxide (ZnO) is a promising semiconductor material due to its pyroelectric properties, stemming from noncentrosymmetric wurtzite crystal structure, enabling the pyro-phototronic effect that enhances response speed. Properties of ZnO can be tailored via alloying and doping. Thus, this work explores thin layers of ZnCdO:Eu random alloys grown by molecular beam epitaxy on silicon substrates with varying Cd content. The study shows that doping with Eu notably affects growth kinetics, promoting a strong [0001] orientation preference. Moreover, photoluminescence measurements confirm the successful incorporation of Eu^3+^ ions into the structure. Electrical measurements show that the introduction of Cd eliminates the problem of Schottky barrier formation at the ZnO/Au interface. The *n*-ZnCdO:Eu/p-Si junctions exhibit rectifying behavior and generate photocurrent across the 370–1150 nm wavelength range without external electrical bias. Utilizing the pyro-phototronic effect, these devices achieved ultrafast response times: rise time below 10 μs and decay time below 5 μs for 405 and 650 nm illumination, placing them among the fastest self-powered oxide-based detectors that do not rely on additional performance-enhancing layers.

## Graphical abstract

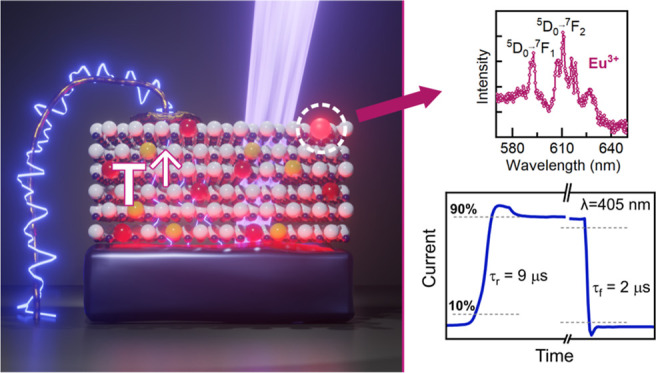

## Introduction

ZnCdO alloy has been drawing the attention of the scientific world for 30 years now.^1^ It is a semiconductor material that offers a tunable bandgap ranging from 3.3 eV (pure ZnO^2^) to 2.18 eV (pure CdO^3^) at room temperature (RT), making it a potential alternative for InGaN in optoelectronic devices.^1^ Moreover, for low Cd contents in ZnCdO, the carrier concentration increases. However, there is a limit for this rise in carrier concentration, when the density of impurities is high enough to effectively scatter electrons, eventually degrading conductance of the material.^4^ As ZnO has a wurtzite structure and CdO crystallizes in a rock salt phase in normal conditions, obtaining good quality structures in case of ZnCdO remains an issue.^1^ Despite that, ZnCdO is being presented as a transparent conductive oxide^1^ or as a candidate for an active material in devices such as light-emitting diodes.^5^ In the latter application, ZnCdO is often used as a well material in multiple quantum well systems with ZnO serving as the barrier layer.^5^ Nevertheless, obtaining a sharp interface in these quantum structures is challenging, as Cd demonstrates tendencies to diffuse into adjacent layers.^6^

Recent years have brought data concerning various aspects of ZnCdO. It has been presented as a digital alloy in the form of a short-period {ZnO/CdO} superlattice, offering an alternative to random alloys.^7–9^ Additionally, ZnCdO doped with Co^10^ or Ti^11^ has been investigated. Regarding doping, pure ZnO doped with various rare-earth elements (REs), such as Eu, La, Tb, and Y, has gained popularity.^12,13^ In response to this growing interest, our previous research described {ZnCdO/ZnO} superlattices doped with Eu grown on Si.^14^

REs are used in ZnO-based materials to modify optical and electrical properties.^15^ They can serve as luminescence centers, enabling f–f and f–d internal orbital transitions.^13^ Specifically, Eu^3+^ ions exhibit a 612 nm transition, making them suitable for potential red LED applications.^16^ A proper concentration of Eu in ZnO has also been reported to induce ferromagnetism at RT.^17^ Moreover, Eu doping contributes to a slight decrease of the optical bandgap,^18^ provides charge carriers, reduces resistivity, and enhances the carrier mobility in ZnO films.^19^ These last three factors are crucial for solar cells and photodetectors, as they facilitate the escape of photogenerated electrons from the junction into the electrical circuit, increasing photocurrent rather than allowing recombination.

Over the past decade, ZnO has found a specific application in photodetectors (PDs) based on the pyro-phototronic effect.^20,21^ This is due to the noncentrosymmetric crystallographic structure of wurtzite ZnO.^22^ In such devices, ZnO serves as a source of an additional electric field that is generated when the intensity of the incident light changes, causing a temporal variation in temperature. This field can be beneficial in photodetectors as it helps to separate photogenerated carriers, resulting in higher photocurrent^22^ and shorter response times.^23^ However, most published studies focus on PDs with pure ZnO,^20,21^ with only a few exploring structures doped with elements such as Ga,^24^ Fe,^25^ and halogens.^26^

In this research, ZnCdO:Eu thin films grown on Si via plasma-assisted molecular beam epitaxy (PA-MBE) are investigated. The introduction of the Eu dopant was performed in situ as an alternative to ion implantation,^27,28^ a commonly used method for incorporating REs that, however, causes greater damage to the crystal lattice. The impact of the Cd content on lattice strain and vibrational properties is analyzed. We demonstrate that the ZnCdO:Eu/Si system can be utilized as a self-powered broadband photodetector operating without applying an external voltage bias in the spectral range of 380–1150 nm. In the investigated devices, Si serves as the absorber, while the ZnO alloy layer forms a p–n junction with Si and enables the pyro-phototronic effect. For self-powered photodetectors, one of the key parameters determining performance is the built-in electric field, *E*_bi_, which separates the photogenerated carriers. According to the literature, Eu incorporation leads to an increase in carrier concentration (and thus the built-in field) and mobility,^19^ facilitating rapid and efficient operation of the photodetector. In addition, Cd, when introduced in low concentrations, can also increase carrier concentration.^4^ The influence of Eu and Cd is schematically shown in Figure 1a. Importantly, from the perspective of future studies, Cd incorporation offers the possibility of tuning the bandgap of the ZnO alloy, thus enabling modulation of the spectral response range of the photodetector. *E*_bi_ is further enhanced by the pyro-phototronic effect, while the temperature of the device is increasing during the light pulse leading to an enhanced recorded signal (cf. Figure 1b).

**Figure 1:**
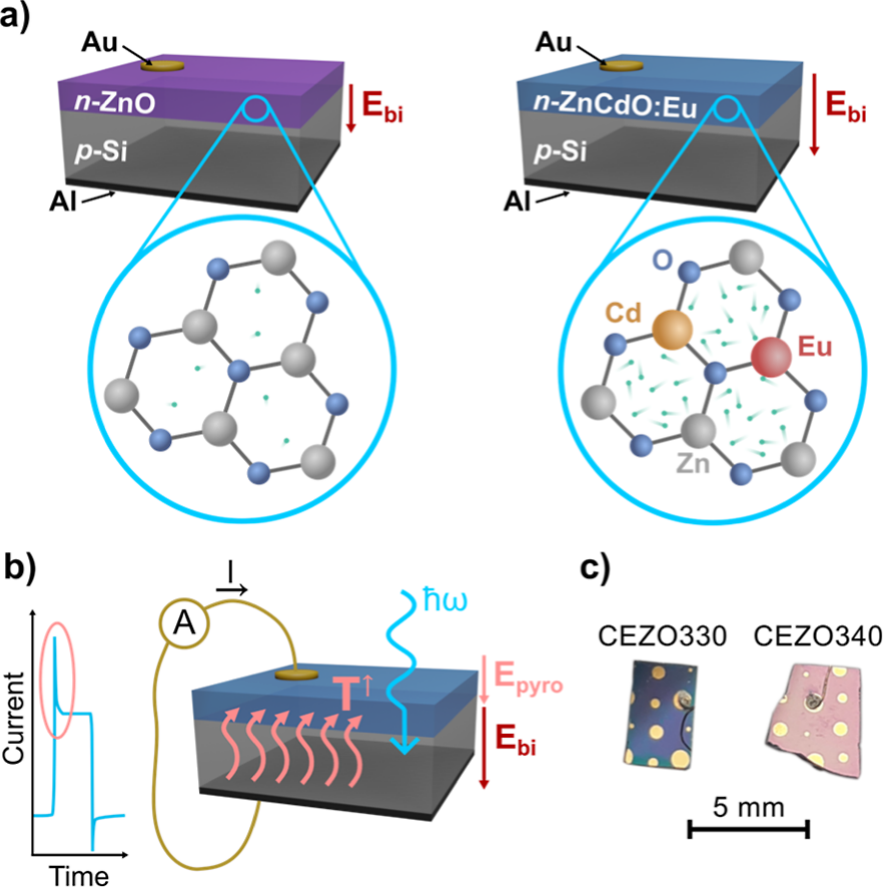
Design principles of the investigated structures. (a) Enhancement of the built-in field *E*_bi_ resulting from the increased carrier concentration introduced by doping. (b) Increased current due to additional electric field generated upon switching on the light source. (c) Images of the selected samples.

In this research, photocurrent generation exceeding the level of 400 mA/W for 700 nm is observed, making ZnCdO:Eu thin films highly attractive for energy-saving optoelectronics. Additionally, the pyro-phototronic effect was detected, contributing to a fast response of the device. The measured rise times were shorter than 10 μs with fall times below 4 μs, classifying these detectors as ultrafast.^29,30^ This research opens a discussion on doped wurtzite-ZnCdO as a potential alternative to pure ZnO in PDs based on pyro-phototronic effects, offering greater flexibility in bandgap and strain engineering.

## Experimental Details

Zn_*x*–1_Cd_*x*_O thin films doped in situ with Eu were grown on p-type Si(001) substrates by plasma-assisted molecular beam epitaxy (Compact 21 Riber). The substrates were degassed in a load chamber at 150 °C for 1 h. Thereafter, the substrate temperature in the growing chamber was raised to 550 °C for 10 min and then reduced to the growth temperature 380 °C. The radio frequency (RF) cell was used to generate oxygen plasma with a power of 400 W and an oxygen flow of 3 mL/min. The temperature of the Zn and Eu effusion cells was fixed at 573 °C (flux: 9.6 × 10^–7^ Torr) and 440 °C (flux: 0.4 × 10^–9^ Torr), respectively. Different Cd concentrations in the films were achieved by changing the temperature of the cadmium effusion cell (320 °C, 330 and 340 °C) and hence the flux (1.2 × 10^–8^ Torr, 3.0 × 10^–8^ Torr, and 3.9 × 10^–8^ Torr, respectively). As a result, four structures were obtained: one ZnO:Eu (EZO) sample and three Zn_*x*–1_Cd_*x*_O:Eu (CEZO) samples: CEZO320, CEZO330, and CEZO340 (cf. Figure 1c), where the numbers correspond to the temperature of the cadmium effusion cell. Thicknesses of the thin films were ∼345 nm, ∼335 nm, ∼445 nm, and ∼445 nm for EZO, CEZO320, CEZO330, and CEZO340, respectively. Small fragments of each sample underwent rapid thermal processing (RTP) for 5 min at 700 °C in an oxygen atmosphere. AccuThermo AW610 from the Allwin21 Inc. system was used for RTP. To enable electrical measurements, Au contacts were sputtered on the thin films, while Al contacts were deposited on Si substrates.

CAMECA IMS6F system was utilized for secondary ion mass spectrometry (SIMS). A Hitachi SU-70 scanning electron microscope (SEM) coupled with a Thermo Fisher Scientific Energy Dispersion X-ray spectrometer with a Li-drift silicon X-ray detector and the Noran System7 were used to study the morphology of samples. The microscope was also equipped with a Gatan MonoCL3 cathodoluminescence (CL) system and a liquid helium cooled cryo-stage to investigate the local optical properties of samples at low temperatures (∼5 K). X-ray diffraction (XRD) measurements were performed using a high-resolution Panalytical X’Pert Pro MRD diffractometer equipped with Cu Kα_1_ radiation, hybrid 2-bounce Ge(220) monochromator, 3-fold Ge(220) analyzer in front of a proportional detector or Soller slits in front of a Pixcel detector. In this work, θ/2θ scans were measured in low angle resolution mode (Soller slits and Pixcel detector). Moreover, high-resolution X-ray diffraction (HRXRD) reciprocal space maps (RSMs) were collected to obtain accurate values of the lattice parameters of the samples.

To collect Raman spectra, a HORIBA Jobin Yvon T64000 system was used. It was configured for backscattering geometry and operated in a single subtractive mode. The spectrometer was working with 0.1 mm slits, resulting with 0.5 cm^–1^ spectral resolution. The samples were excited with a 532 nm semiconductor laser. A CCD detector was used for scattering light detection. Peak positions of Raman modes were found by fitting the data with the Lorentz functions. Raman spectral measurements were repeated 15 times for different spots on each of the samples. Photoluminescence spectra were measured using a Horiba Jobin-Yvon Fluorolog-3 spectrofluorometer equipped with a 450 W xenon lamp and a Hamamatsu R928P photomultiplier for UV and visible range detection.

Current–voltage (*I*–*V*) characteristics were collected at RT using myDLTS software^31^ and the following hardware: Keithley 2601A *I*–*V* source meter and Zurich Instruments MFIA Impedance Analyzer, which were also used to perform RC time constant measurements. During current–time (*I*–*t*) characteristics measurements, the samples were illuminated using 405 and 650 nm lasers. A Bentham PVE300 Photovoltaic Device Characterization System was used to record the responsivity spectra. These results were accompanied by reflectance spectra measured using a Jasco V770 UV–visible/NIR spectrophotometer. An *I*–*V* curve tracer with a PET Solar Simulator (#SS100AAA) were utilized to collect dark and light *I*–*V* data (AM1.5G conditions, 1000 W/m^2^ light intensity, 25 °C ambient temperature).

## Results and Discussion

### Structural Properties

The collected SIMS signals for Cd and Eu were at the verge of system detection possibilities. The concentration of Cd reached a maximum value of 5 × 10^18^ cm^–3^ for the CEZO340 sample (cf. Figure S1). In contrast, the Eu signal could not be quantified reliably, as the SIMS detection limit for Eu in ZnO is approximately 10^17^ cm^–3^ (cf. Figure S2). The sensitivity of the EDX (energy-dispersive X-ray spectroscopy) technique was also insufficient to provide accurate estimates of Cd and Eu contents (Figure S3). However, the presence of Eu^3+^ ions is unambiguously confirmed by the PL data presented later in this section.

X-ray diffraction patterns on a logarithmic scale are shown in Figure 2a. The results confirm the growth of wurtzite ZnO. All the spectra exhibit dominant 0002 and 0004 ZnO peaks, corresponding to the [0001] orientation of the wurtzite ZnO phase. The remaining ZnO-related signals are relatively weak. The XRD scans demonstrate a strong preferential growth of ZnO along the [0001] direction when doped with Eu. Similar behavior was observed in our previous study regarding Eu-doped {ZnCdO/ZnO} superlattices grown on Si by Lysak et al.^32^ The literature describing Eu-doped ZnO films and nanostructures reports comparable orientation preferences across various substrates.^19,33,34^ Additionally, a very sharp peak is observed at 33° (Figure 2a). It comes from the 200 Si quasi-forbidden reflection. These quasi-forbidden reflections are registered because the incident X-ray beam is formed by a hybrid 2-bounce monochromator and the beam is divergent (12 arcsec). The existence of such reflections was first described by Renninger over 60 years ago.^35^

**Figure 2:**
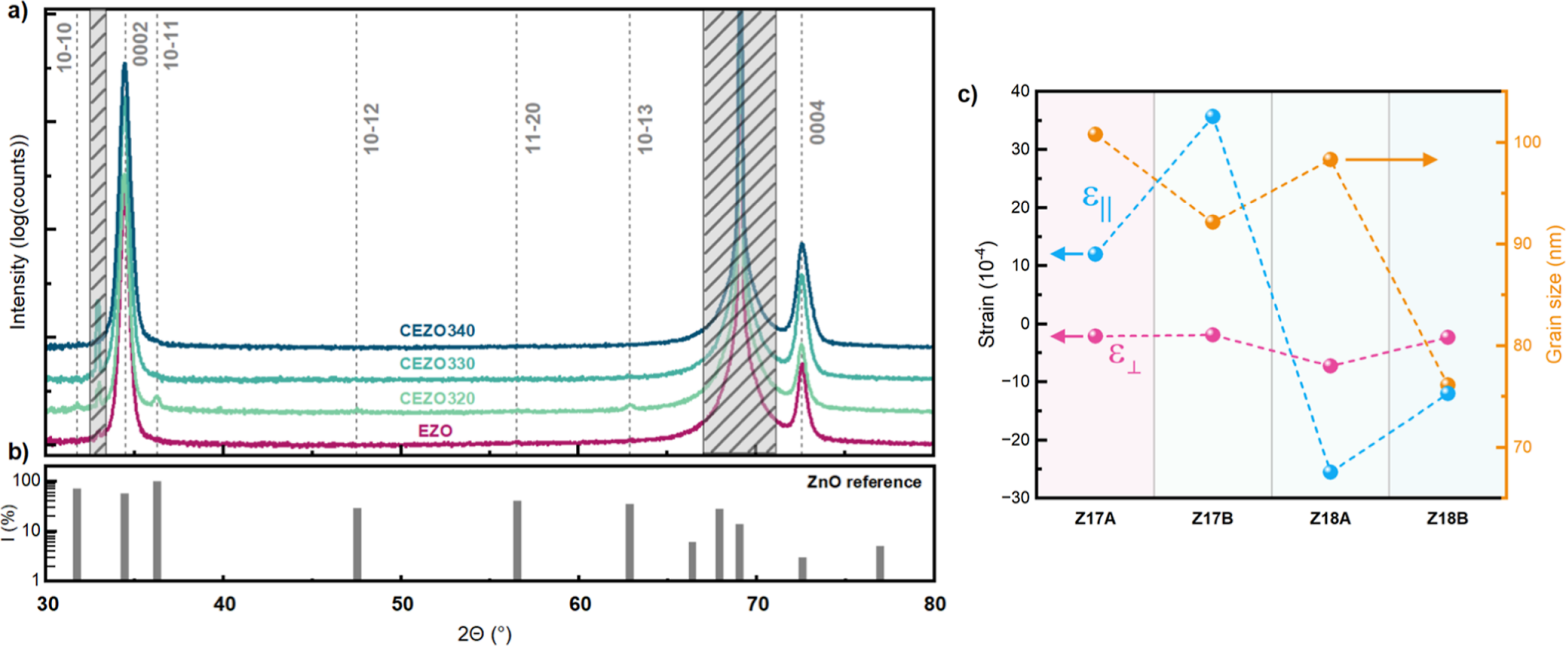
(a) X-ray diffraction patterns for ZnO:Eu and ZnCdO:Eu films in the logarithmic scale. Si-related range is hatched. The positions of ZnO-related peaks are marked according to the JCPDS 00-005-0664 card. (b) Relative intensities of XRD peaks in ZnO reference according to JCPDS data. (c) Calculated out-of-plane and in-plane strain, ε_⊥_ and ε_∥_, respectively, grain size based on XRD data—dashed lines added to guide the eye.

To calculate the accurate values of lattice parameters, HRXRD RSMs were registered (shown in Figure S4 in the Supporting Information). From the maxima of HRXRD signals in RSMs, the interplanar distance *d*_*hkl*_ (where *hkl* denotes the Miller indices) was calculated using Bragg’s law:^32^

1 $$d_{hkl}=\frac{m\lambda }{2\sin\theta_{hkl}}$$

where *m* is the order of reflection (*m* = 1), λ denotes the X-ray wavelength (λ = 1.54056 Å), and Θ is the Bragg diffraction angle obtained from the peak maximum in the HRXRD reciprocal space map.

For the wurtzite structure, the relation between *d*_*hkl*_ and the lattice parameters *a* and *c* is determined by the quadratic equation as follows:^32^

2 $$\frac{1}{d_{hkl}^{2}}=\frac{4}{3}\cdot \frac{h^{2}+hk+k^{2}}{a^{2}}+\frac{l^{2}}{c^{2}}$$

To extract both *a* and *c* lattice parameters, the ZnO 0002 symmetrical reflection and ZnO −1–124 asymmetrical reflection were measured. The HRXRD RSMs of the ZnO 0002 reflection, with *hkl* = 002, were used for determination of the *c* lattice parameter. By combining eqs 1 and 2 under these conditions, the following relation can be derived:

3 $$c=\frac{\lambda }{\sin\theta }$$

Using the obtained *c* lattice constant, the out-of-plane strain component can be calculated as follows:^32^

4 $$\varepsilon_{⊥}=\frac{c-c_{0}}{c_{0}}$$

where *c*_0_ is the reference lattice constant of relaxed ZnO bulk material (*c*_0_ = 5.2066 Å, according to the JCPDS database).

Next, the ZnO −1–124 asymmetrical reflection (with *hkl* = −1–14) was used to determine the in-plane *a* lattice parameter. By combining eqs 1–3, the *a* lattice parameter can be derived:

5 $$a=\sqrt{\frac{4\cdot \left(h^{2}+hk+k^{2}\right)\cdot d^{2}\cdot c^{2}}{3\cdot \left(c^{2}+d^{2}\cdot l^{2}\right)}}$$

Using the obtained *a* lattice constant, the in-plane strain component is then calculated as^32^

6 $$\varepsilon_{∥}=\frac{a-a_{0}}{a_{0}}$$

where *a*_0_ is the reference lattice constant of relaxed ZnO bulk material (*a*_0_ = 3.2498 Å, according to the JCPDS database).

The grain size *D* of the ZnCdO:Eu films was calculated by using the Lorentzian component of the diffraction line broadening (FWHM, β_*L*_in radians), obtained from fitting the diffraction peaks with a Voigt function. The grain size is determined according to the Debye–Scherrer formula:^36^

7 $$D=\frac{k\lambda }{\beta_{L}\cos\theta }$$

where *k* is a constant that equals 0.94 for spherical particles.^36^ Parameters calculated using eqs 4, 6, and 7 are depicted in Figure 2c. Cd^2+^ and Eu^3+^ ions have similar ionic radius −0.097 and 0.095 nm, respectively^32^—both significantly larger than that of Zn^2+^, which totals 0.074 nm.^37^ Hence, changes in the strain with doping are expected. According to our results, the samples display near-zero strain along the growth direction (out-of-plane), thereby minimizing any piezo-induced modulation of the internal electric field. The calculated grain sizes ranged from 75 to 105 nm.

Raman spectroscopy was applied to analyze the vibrational properties of the ZnCdO:Eu films. In this case, to obtain additional information regarding crystal lattice behavior, small fragments of the samples underwent rapid thermal processing (RTP). As shown in Figure 3a, the spectra were dominated by Si-related modes, with the 521 cm^–1^ LTO­(Γ) mode^38^ being the most intense. The peaks having origins in the Si substrate are marked with asterisks. A detailed description of the Si bulk Raman spectrum can be found in our previous study.^14^ The ZnO *E*_2_^low^ and *E*_2_^high^ modes were found at 99 and 437 cm^–1^, respectively. Contrary to Eu-doped {ZnCdO/ZnO} superlattices,^14^ no additional modes were observed except for the CEZO340 as-grown sample. In this case, an asymmetrical broadening of the Si LTO­(Γ) mode was recorded (Figure 3a, marked with an orange circle). This is probably due to a defect-related band emerging in the ZnO Raman spectra, possibly attributed to the A_1_(LO)^39^ or the mixed qA­(E)_1_ mode.^14^ The contribution of second-order Raman modes cannot be excluded.^40^ The appearance of these modes is a sign of a disordered ZnO lattice, possibly due to zinc interstitials and oxygen vacancies.^39,40^ Hence, the increase in the Cd concentration promotes the formation of these defects. Zinc interstitials, oxygen vacancies, and their complexes are known to act as donor centers, providing additional free carriers to the material and potentially influencing the electrical and photoresponse characteristics of the investigated heterostructures.^41^

**Figure 3:**
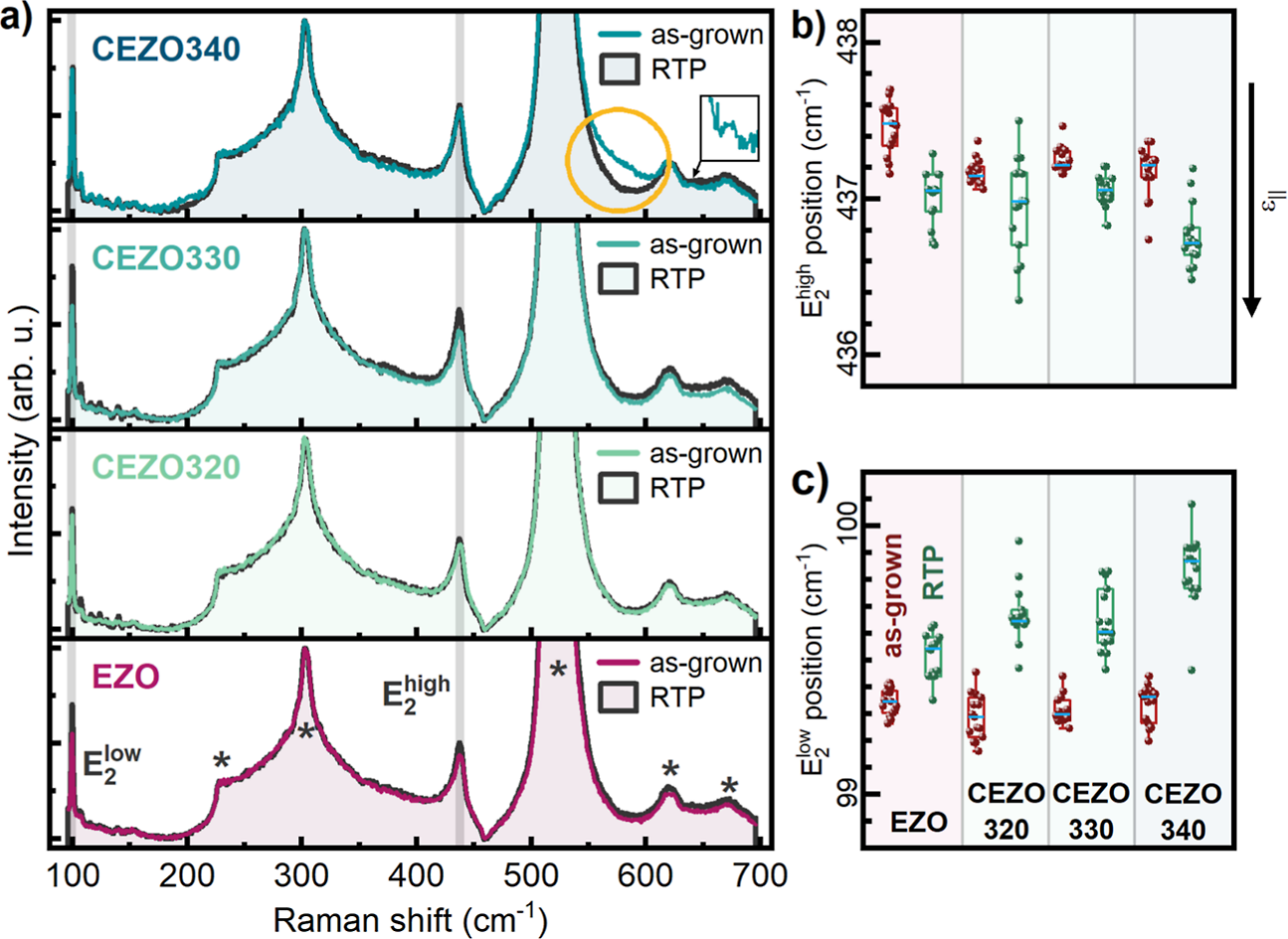
(a) Raman spectra collected for as-grown and annealed (RTP) samples. The spectra were normalized to the 303 cm^–1^ Si mode. Comparison of the peak positions of (b) *E*_2_^high^ mode with corresponding in-plane strain, ε_∥_, and (c) *E*_2_^low^ mode for the studied samples. Red data points correspond to as-grown structures, while green data points represent structures after RTP. Blue lines inside the boxes indicate median values. Each box contains 50% of the data, ranging from the first and third quartile.

Moreover, a weak 640 cm^–1^ peak can be spotted, generally identified as an ‘additional mode’,^42^ or more specifically as the TA + *B*_1_^high^ silent mode.^14^ This band is commonly observed in ZnO-based structures doped with various elements, such as N, Eu, Ga, and Fe.^14,42,43^ Both of these features disappear after annealing, a behavior also observed in Eu-doped {ZnCdO/ZnO} SLs.^14^

An analysis of the shifts of Raman modes provides insight into the strain present in the structure. The *E*_2_^high^ ZnO mode is known to shift linearly with increasing in-plane strain, ε_∥_.^14,44^ Specifically, a redshift of the *E*_2_^high^ mode from its reference unstrained position indicates tensile strain, whereas a blueshift suggests compressive strain.^14,44^ The measured *E*_2_^high^ peak positions are collected in Figure 3b. Annealing results in a redshift of the *E*_2_^high^ mode, indicating that the variation in in-plane strain results in lattice expansion. This effect was also observed in Eu-doped {ZnCdO/ZnO} SLs.^14^ In contrast, the *E*_2_^low^ ZnO mode shows the opposite behavior (cf. Figure 3c), where annealing causes a blueshift of this peak.

Figure 4 shows cathodoluminescence (CL) spectra measured for the as-grown structure and the fragments of the samples annealed in oxygen by the RTP method. The most intensive peak detected at about 3.3 eV most probably comes from donor-bound exciton states D^0^X.^45^ A less intensive peak related to free exciton (FX) emission is visible at about 3.343 eV, as well. Moreover, the defect-related broad band emission can be observed in the 2.0–3.0 eV region (visible spectral range). This band emerges for both as-grown and annealed samples. In the case of the sample with the highest Cd concentration (CEZO340), a stronger defect-related emission is observed. The origin of this band is most likely a result of native defects in the ZnCdO lattice,^46^ supporting previous observations from Raman spectroscopy (cf. Figure 3a). Similarly to the results obtained by that method, the CL spectra show that in the case of the CEZO340 structure, RTP reduces the concentration of defects, as evidenced by the disappearance of the broad emission band, as shown in Figure 4.

**Figure 4:**
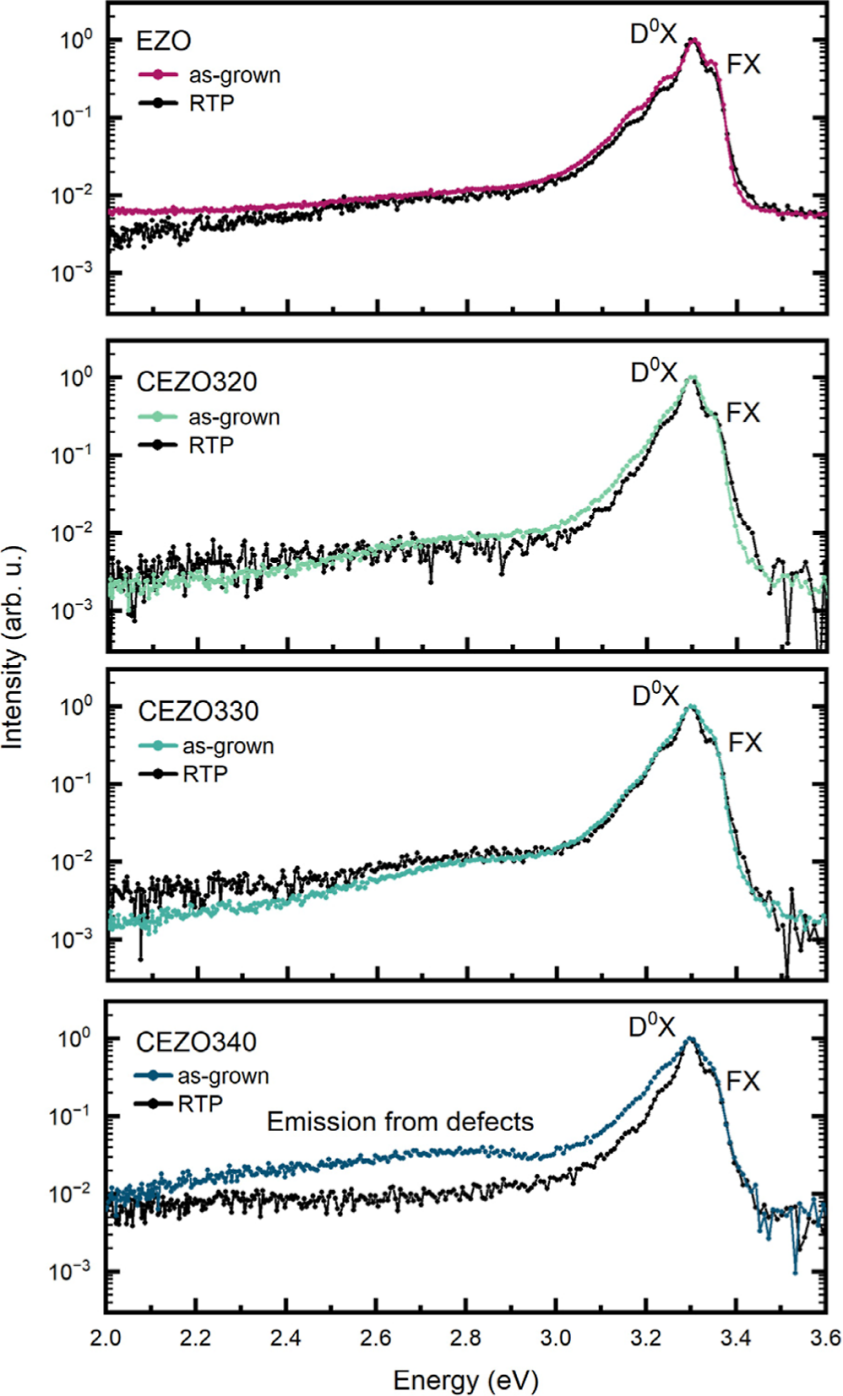
Low-temperature normalized cathodoluminescence spectra of as-grown and annealed samples.

The photoluminescence spectra shown in Figure 5a confirm the successful doping of the material with Eu^3+^ ions that can provide additional carriers to the material. Upon excitation at 260 nm, characteristic emission peaks related to intra 4f transitions in Eu^3+^ ions are observed. The spectra exhibit typical emission peaks located at 592 nm (^5^D_0_ → ^7^F_1_ transition) and 611 nm (^5^D_0_ → ^7^F_2_ transition). Figure 5b represents the possible energy transfer mechanism occurring during the experiment. Upon absorption of 260 nm (4.77 eV) photons, electrons are excited from the valence band (*E*_v_) to energies above the conduction band (*E*_c_). Then, they relax to form *E*_c_. Subsequently, nonradiative transfer of these electrons from the ZnO host to the ^5^D_0_ level of Eu^3+^ ions may occur, either directly or via defect levels, such as Eu_Zn_ substitutions or zinc interstitials. This process is followed by the radiative ^5^D_0_ → ^7^F_2_ and ^5^D_0_ → ^7^F_1_ transitions giving rise to the characteristic Eu^3+^ emission.^47^ Crystal-field splitting is observed in the Eu^3+^ emission lines. It is well established that the local symmetry of Eu^3+^ sites is reflected in the features of the ^5^D_0_ → ^7^F_*J*_ (*J* = 0, 1, 2, 3, and 4) transitions. Occupation of low-symmetry sites leads to splitting of these emission peak, yielding a single line for the ^5^D_0_ → ^7^F_0_ transition, three for ^5^D_0_ → ^7^F_1_, and five for ^5^D_0_ → ^7^F_2_.^48,49^

**Figure 5:**
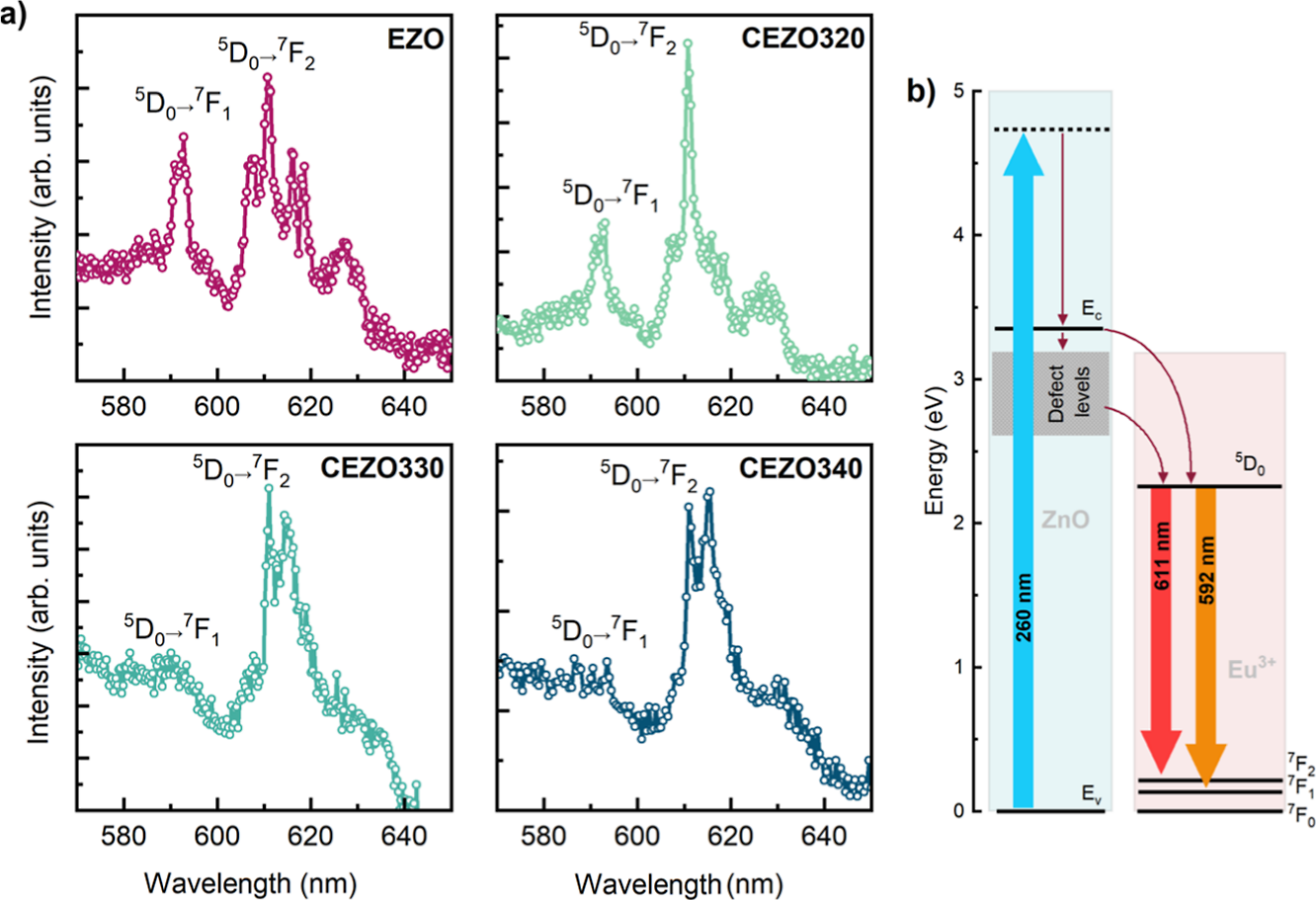
(a) Photoluminescence spectra displaying emission from Eu^3+^ ions and (b) schematic representation of the energy transfer process observed during the measurement, based on Zhang et al.^47^ The samples were excited with a 260 nm lamp source.

### Electrical Properties and Photodetection

The current–voltage measurements indicate difficulties in obtaining a rectifying junction for structures without Cd and those with the lowest Cd content, namely, EZO and CEZO320 (cf. Figure 6a and the inset in Figure 6b, respectively). This is due to the formation of an undesirable potential barrier, evidenced by a sudden increase of current in reverse bias at −1 V. The application of Au contacts directly onto ZnCdO:Eu thin film may contribute to this behavior, as Au can form a Schottky junction with ZnO.^50^ However, some reports describe devices with Au electrodes applied on ZnO that do not feature this issue.^51,52^ The turn-on voltages, *V*_on_, for these samples were estimated to be at the level of ∼2.0 V, which aligns with values commonly reported in the literature, typically ranging from 1 to 2.5 V.^53,54^ The rectifying factor (RF) at ± 4 V totals 0.2 and 25 for EZO and CEZO320, respectively.

**Figure 6:**
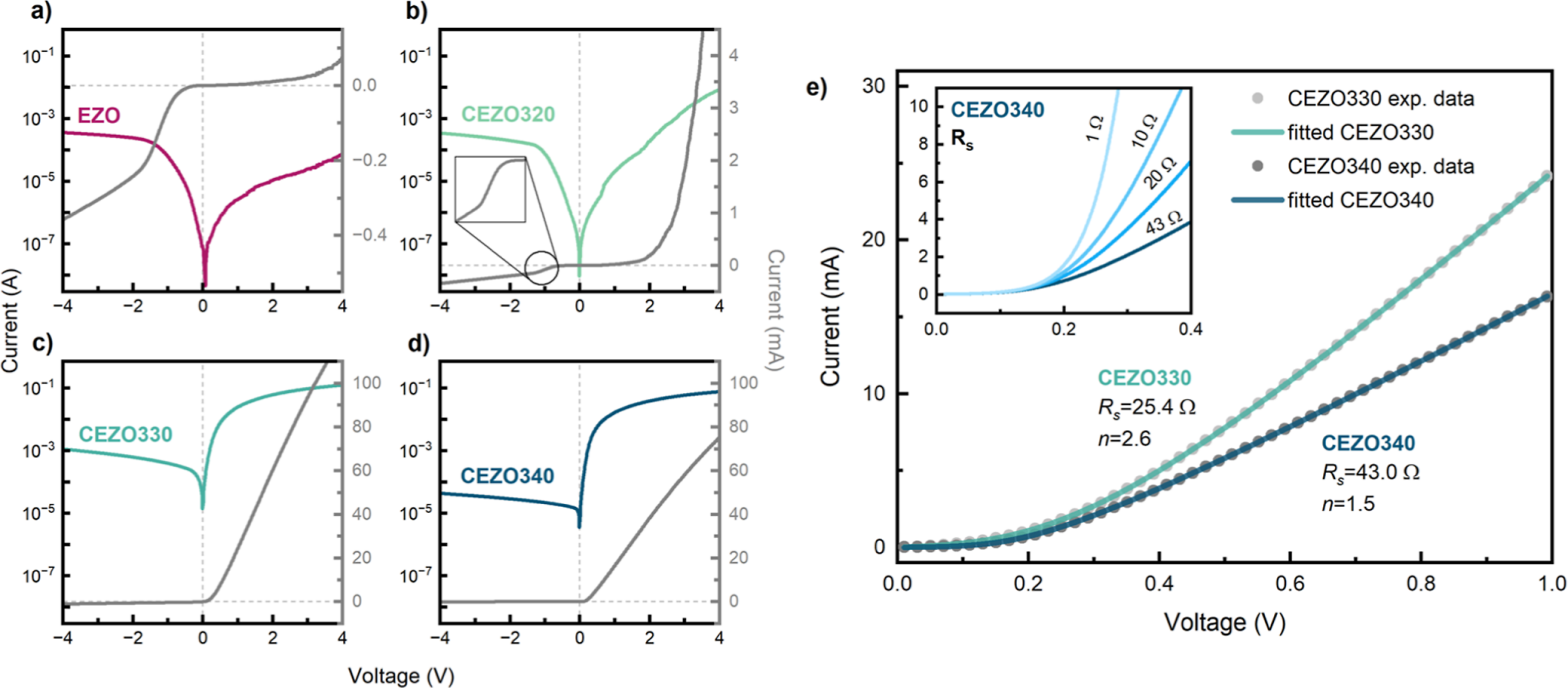
Dark current–voltage curves of samples (a) EZO, (b) CEZO320, (c) CEZO330, and (d) CEZO340 measured at room temperature. Colored curves are presented in a semilogarithmic scale (left axis), whereas the gray curves are on a linear scale (right axis). (e) Fitted experimental data and the extracted electrical parameters. The inset illustrates the influence of series resistance, *R*_s_, on the low-voltage *I*–*V* curve of the CEZO340 sample.

Samples 18A and 18B (cf. Figure 6c,d) do not seem to encounter the problem of the additional potential barrier at the metal/semiconductor interface. There are several mechanisms that may contribute to the observed behavior, including doping-induced Schottky barrier reduction,^55^ changes in morphology or high local electron concentration enabling tunneling transport, resulting in ohmic-like behavior.^56^ Further studies are required to clarify this issue.

RF values of CEZO330 and CEZO340 junctions calculated for ±4 V were found to be around 110 and 1700, respectively. This difference is mainly sourced from the current that flows through junctions in the reverse bias, as in CEZO340 structure, it is over one order lower than in CEZO330. The *I*–*V* curves of both CEZO330 and CEZO340 samples exhibit nearly zero turn-on voltage (∼0.2 V). A turn-on voltage *V*_on_ = 0.4 V for the ZnO/Si junction was previously obtained by Ghosh and Basak.^57^ Similarly, CdO/Si structures are also known to exhibit such low *V*_on_ values.^58,59^ However, these examples generally suffer from poor rectification, particularly when compared to the CEZO340 structure, which exhibits RF exceeding 50 at just 0.21 V. An exceptionally low *V*_on_, combined with a high RF, may be beneficial in terms of the use of such structures in current rectifiers operating at low voltages.

Nearly linear *I*–*V* characteristics for voltages over 0.2 V indicate a high series resistance of the junction.^60^ This deviation from the ideal exponential *I*–*V* behavior is well-described by the single exponential model of a solar cell based on a p–n junction that takes into consideration both series resistance (*R*_s_) and shunt resistance (*R*_sh_):^60,61^

8 $$I=I_{0}\left(e^{\left(V+IR_{s}\right)/nV_{th}}-1\right)+\frac{V+{IR}_{s}}{R_{sh}}-I_{ph}$$

where *I*_0_ is the saturation current, *V*_th_ is the thermal voltage that equals to *k*_B_ *T*/*q*, where *q* is the electric charge, *k*_B_ is the Boltzmann constant, *T* denotes the temperature, and *n* denotes the ideality factor, *I*_ph_ is the photogenerated current. The exact solution of eq 8 is expressed by^60^

9 $$I=\frac{V}{R_{s}+R_{sh}}+\frac{nV_{th}}{R_{s}}\cdot W\left\{\frac{R_{s}I_{0}R_{sh}}{nV_{th}\left(R_{s}+R_{sh}\right)}\exp\left[\frac{R_{sh}\left(R_{s}I_{ph}+R_{s}I_{0}+V\right)}{nV_{th}\left(R_{s}+R_{sh}\right)}\right]\right\}-\frac{R_{sh}\left(I_{0}+I_{ph}\right)}{R_{s}+R_{sh}}$$

The junctions that did not exhibit issues related to Au/Zn­(Cd)­O Schottky contact formation were fitted by using eq 9 and OriginPro software to extract the electrical parameters. The results are presented in Figure 6e (for *I*_ph_ = 0). In both cases, the shunt resistance was extremely high (>1 MΩ), indicating the absence of relevant alternative current channels in the dark. The *R*_s_ totaled 25.4 Ω for the CEZO330 sample and 43.0 Ω for the CEZO340 sample. The impact of *R*_s_ on device behavior is presented for the CEZO340 structure in the inset of Figure 6e. These curves were generated using eq 9 with parameters extracted from the experimental data, followed by manual adjustment of *R*_s_ values. The ideality factor, *n*, informs us about current transport mechanisms within the junction. For the CEZO330 sample, the extracted value of *n* = 1.5 lies within the range of 1–2, suggesting that current transport is governed by diffusion and generation–recombination processes.^62^ In contrast, for the CEZO340 sample, *n* exceeds 2, indicating that other mechanisms—such as carrier trapping or tunneling^62^—need to be considered.

All of the samples generate photocurrent, as shown in Figure 7a,b. The responsivity of a detector is defined as

10 $$R=\frac{I_{light}-I_{dark}}{P}$$

where *I*_light_ is the current measured under illumination, *I*_dark_ denotes the current measured in the dark. These values were obtained without applying an external bias. *P* represents the power of the incident light. As shown in Figure 7c, the extracted *R* was measured locally rather than globally. The light spot had a square shape (1.15 × 1.15 mm) and was positioned to illuminate the Zn­(Cd)­O:Eu layer between the Au contacts near the active Au contact connected to a Au wire. The structures convert photons with wavelengths longer than 370 nm, which corresponds to the 3.35 eV bandgap of ZnO. Photons of higher energy likely do not contribute to photocurrent due to strong absorption near the surface. It is worth noting that the incorporated Cd concentration in CEZO samples is insufficient to significantly redshift the optical bandgap and hence the detection limit. The long-wavelength limit, around 1150 nm, is strictly imposed by the bandgap of the Si absorber. This makes the ZnCdO:Eu/Si system suitable for broadband light detection applications. However, extending the response further into the short-wave infrared region would require a hybrid design integrating narrower-bandgap materials. The visible-range photocurrent arises mainly from band-to-band absorption in Si, followed by carrier separation at the heterojunction interface. Defect-assisted absorption in ZnCdO can possibly contribute, as well. Eu incorporation improves device performance indirectly by increasing the carrier concentration and mobility, thereby strengthening the built-in electric field and enhancing carrier separation. At low concentrations, Cd also contributes to an increased carrier concentration. Both dopants may additionally influence defect states and recombination dynamics. Thus, Eu^3+^ acts primarily as a spectroscopic marker of successful doping and a modifier of the electronic structure, rather than a direct contributor to photocurrent via its ^5^D_0_ → ^7^F_2_ emission. These combined effects result in the efficient visible-range photoresponse observed in Figure 7b. Samples CEZO330 and CEZO340 exhibit the highest responsivity. For CEZO330, *R* remains around 100 mW/A in the 450–1050 nm wavelength range. The responsivity of CEZO340 is even higher and closely follows the shape of the spectrum of the Si reference detector. The differences in *R* values between these two samples cannot be explained by differences in optical reflection of the film, as the reflection spectra of both ZnCdO:Eu layers were nearly identical, as shown in Figure 7d. The oscillations observed in the measured spectra originate from thin-film interference.

**Figure 7:**
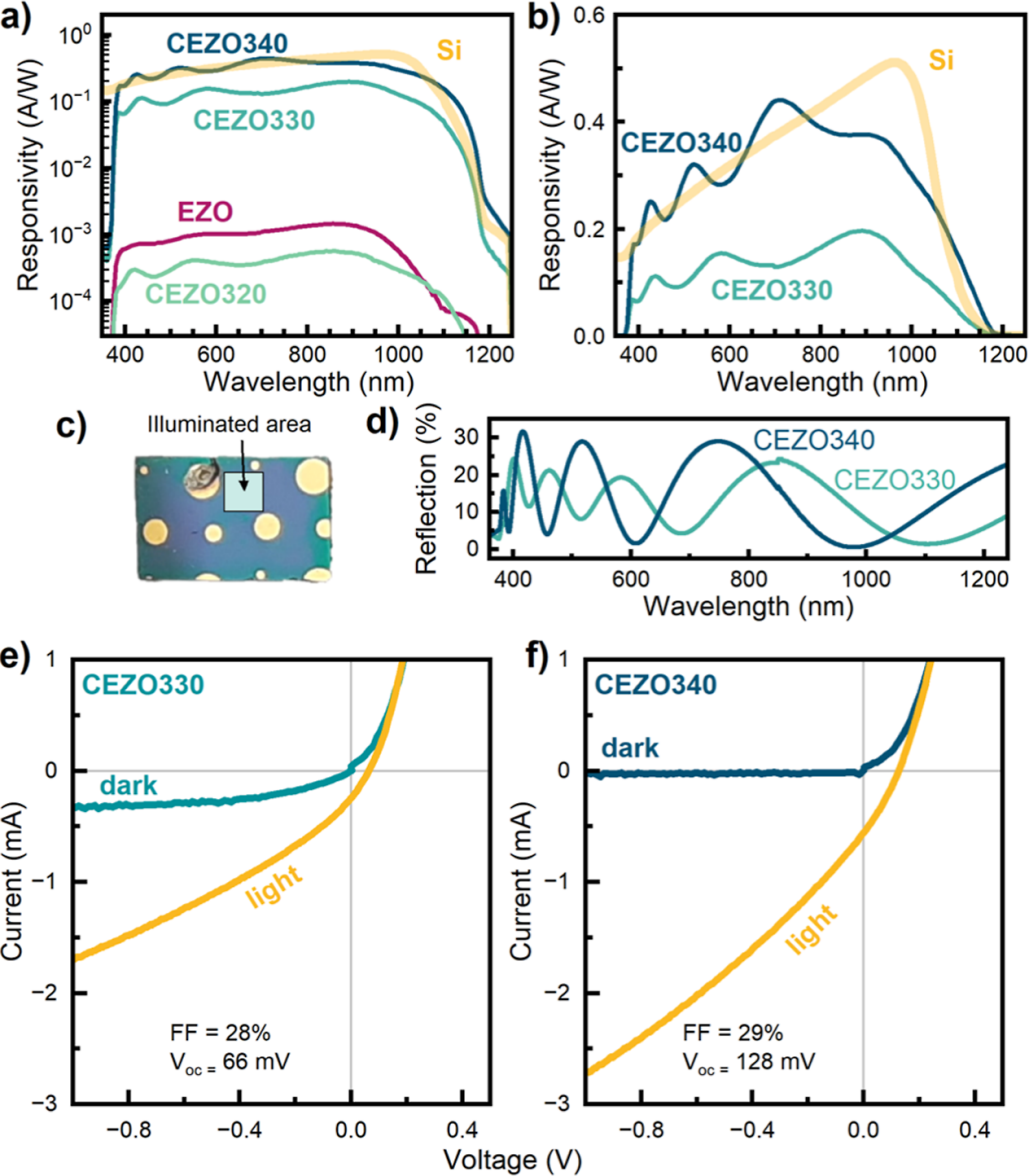
Photocurrent generation spectra of the investigated ZnCdO:Eu/Si junctions compared to a standard Si photodetector in (a) semilogarithmic and (b) linear scale. (c) Schematic illustration of the illuminated sample surface area. (d) Reflectance spectrum measurements. Dark and light *I*–*V* characteristics of samples (e) CEZO330 and (f) CEZO340 measured with the PET Solar Simulator (AM1.5G conditions, 1000 W/m^2^ light intensity, 25 °C ambient temperature).

Light and dark *I*–*V* curves were measured for the structures demonstrating the highest photocurrent generation (CEZO330 and CEZO340), as presented in Figure 7e,f. These measurements are combined with the calculated fill factor (FF) and open-circuit voltage (*V*_oc_). The relatively low FF and *V*_oc_ values, together with the steep character of light *I*–*V* curves in reverse bias, can mainly be explained by a high series resistance.

To estimate the response times of the samples, current–time (*I*–*t*) curves were measured with the laser modulated at 50 ms intervals. Figure 8a presents the photocurrent response of the CEZO330 sample to a 650 nm laser pulse at various temperatures. In addition to the current arising from the photovoltaic effect (*I*_ph_), a signal attributed to the pyro-phototronic effect is also observed. When the laser is switched on, it induces a localized heating of the structure. As the sample temperature, *T*, changes in time, $\frac{\partial T}{\partial t}\neq 0$ , an additional electric field is induced within the ZnCdO:Eu layer. This results in a spike observed in the *I*–*t* characteristics, marked as “*I*_ph+pyro_ heating”. Similarly, turning the laser off causes a decrease of the sample temperature, and while the condition $\frac{\partial T}{\partial t}\neq 0$ is fulfilled, an electric field of opposite direction is induced, causing the flow of current marked as “*I*_ph+pyro_ cooling”. The 100 Hz current oscillations observed under illumination are attributed to electrical interference originating from the AC power grid and its rectification in the power supply units of the measurement equipment. Both *I*_pyro+ph_ and *I*_ph_ change with the temperature.

**Figure 8:**
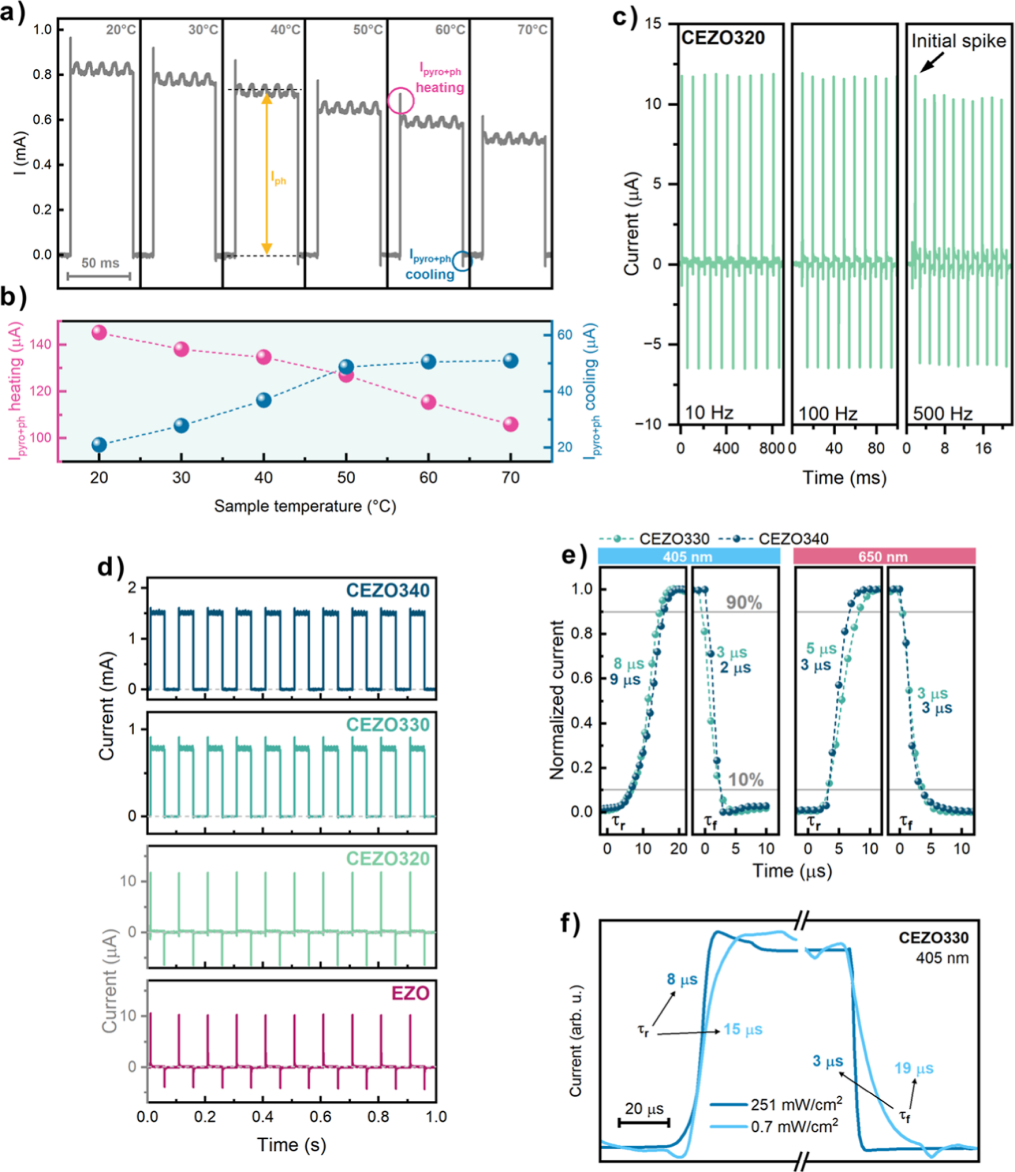
(a) *I*–*t* chart of a single laser pulse measured for the CEZO330 structure at various temperatures (650 nm laser, 540 mW/cm^2^ light intensity). (b) Mean values of the measured *I*_pyro+ph_ during the laser switching on (heating) and off (cooling). (c) Impact of laser pulse frequency on pyro-phototronic effect. (d) Current–time curves of the investigated structures. Samples were illuminated with a 650 nm laser for 50 ms every 100 ms. (e) Zoomed-in *I*–*t* curves illustrating the response times obtained for different wavelengths. (f) Suppression of the pyro-phototronic effect under low-intensity illumination conditions.

The behavior of *I*_pyro+ph_, presented in Figure 8b, confirms that the observed spikes in the transients originate from the pyro-phototronic effect. As can be seen from the data, the *I*_pyro+ph_ heating current decreases, while the *I*_pyr+ph_ cooling current increases with the temperature of the device. A higher sample temperature implies a higher difference in *T* between the sample and the ambient environment (RT). Hence, after turning the laser off, the structure returns to its thermal equilibrium quicker at higher temperatures. For the same reason, the additional heat provided by the laser causes a smaller temperature rise at higher sample temperatures. This explains the observed decrease of the *I*_pyro+ph_ current. The reduction of *I*_ph_ with increasing *T* can be explained by the rise in the saturation current *I*_0_, which is given by equation:^63^

11 $$I_{0}=C\cdot T^{3}\cdot \exp\left(-\frac{E_{g}}{k_{B}T}\right)$$

where *C* is a constant, *E*_g_ is a bandgap.

Another point supporting the pyro-phototronic nature of the observed photodetection is provided by measurements performed at different laser pulse excitation frequencies (Figure 8d). These measurements were carried out for the CEZO320 sample, which exhibits a low-efficiency photovoltaic response, allowing clearer identification of the pyro-phototronic contribution. The selected pulse frequencies were 10, 100, and 500 Hz. The absolute amplitudes of the current spikes observed upon laser illumination were 11.78, 11.45, and 10.69 μA, respectively (averaged over 100 cycles). The gradual decrease in the spike amplitude with increasing frequency is attributed to incomplete thermal relaxation of the sample between consecutive pulses. This effect is particularly evident at 500 Hz. While the initial current spike (*I*_pyro+ph_ heating) reaches the same level as for longer-pulse periods, subsequent spikes show a pronounced reduction. Moreover, the decay of the current spike toward the steady-state photocurrent level is much slower than the pulse period. After the laser is switched off, the sample does not cool to the ambient temperature, as indicated by the current not returning to the dark current level. Consequently, the temperature gradient, and thus the enhancement originating from the pyro-phototronic effect, decreases at higher light pulse frequencies.

*I*–*t* transients measured under periodic 650 nm laser illumination for the studied samples are combined in Figure 8d. As expected from the photocurrent spectra, samples EZO and CEZO320 display almost negligible photocurrent generation, with only peaks related to the pyro-phototronic effect visible. In contrast, both the photovoltaic and pyro-phototronic effects occur in samples CEZO330 and CEZO340.

Figure 8e shows zoomed-in *I*–*t* curves for samples CEZO330 and CEZO340 under 405 and 650 nm laser illumination, respectively, with rise (τ_r_) and fall (τ_f_) times estimated. The photocurrent was normalized to facilitate comparison between the samples. The rise time τ_r_ is defined as the time required for the photocurrent to increase from 10% to 90% of its maximum value, while the fall time τ_f_ represents the time needed for the current to drop from 90% to 10%. For the 405 nm blue laser, τ_r_ and τ_f_ totaled 8 and 3 μs for the CEZO330 sample and 9 and 2 μs for CEZO340. Under 650 nm red laser illumination, τ_r_ and τ_f_ changed to 5 and 3 μs for CEZO330, and 3 and 2 μs for CEZO340. These measurements were performed using the maximum available laser powers of 251 mW/cm^2^ for the 405 nm laser and 540 mW/cm^2^ for the 650 nm laser. To determine whether light intensity has a relevant impact on τ_r_ and τ_f_, additional tests were conducted on the CEZO330 sample, with light intensity modulated via optical filters. When illuminated with the 405 nm laser, τ_r_ and τ_f_ remained constant at 8 and 3 μs, respectively, across the 20–251 mW/cm^2^ range. At a lower laser power of 9 mW/cm^2^, τ_r_ increased to 14 μs and τ_f_ increased to 12 μs. Under even weaker illumination (0.7 mW/cm^2^), the impact of the pyro-phototronic effect was no longer evident—τ_r_ and τ_f_ increased to 15 and 19 μs, respectively, and no *I*_pyro+ph_ spikes were observed (cf. Figure 8f). In the case of the 650 nm laser, τ_r_ and τ_f_ stayed stable at 5 and 3 μs, respectively, across the 5–540 mW/cm^2^ range. Only at the lowest measured light intensity of 0.75 mW/cm^2^ these times increase to 9 and 10 μs, respectively. Raw transient photocurrent curves used to extract rise and fall times presented in Figure 8e,f are shown in Figure S7.

The superior performance of CEZO340 cannot be attributed solely to improved Ohmic contact formation. Increasing the Cd content modifies the electronic structure of ZnCdO, leading to shifts in band alignment at the ZnCdO/Si interface. These changes affect the barrier height and depletion region width, thereby influencing carrier transport across the junction. Variations in Cd concentration also impact the layer conductivity and series resistance, which are crucial for rectification behavior and response speed. Within the pyro-phototronic framework, optimized junction electrostatics enable more efficient modulation of the interfacial barrier under thermal and optical stimulation. Among the investigated samples, CEZO340—corresponding to the highest Cd content within the studied low-doping regime—exhibits the most favorable balance of these effects, resulting in enhanced responsivity, rectification ratio, and ultrafast response compared to compositions with lower Cd incorporation.

To exclude RC-limited behavior, the series resistance (*R*_s_) and capacitance (*C*) of the equivalent RC circuit were measured using an impedance analyzer with a 100 mV AC test signal at varying frequencies (cf. Figure S5). The resulting RC time constants (τ = *R*_s_·*C*) at 1 MHz were 0.28 and 0.11 μs for CEZO330 and CEZO340, respectively, which are well below the measured rise and fall times of the photocurrent. This indicates that the detector response is not limited by device capacitance, while the pyro-phototronic effect effectively reduces the rise and fall times toward the RC limit of the system.

To evaluate the stability of current generation associated with the pyro-phototronic and photovoltaic effects, durability tests were carried out on the CEZO330 and CEZO340 samples. The results presented in Figure 9 demonstrate that the photovoltaic response of both samples remains stable over a 5 h measurement period, as no significant variation in the signal level is observed. After 5 h of constant illumination, the signal decreases by only about 1% relative to its maximum value. Figure 10 presents cyclic switching endurance tests of self-powered photodetection performed under 10^4^ laser pulse cycles. The data envelope indicates that no signal degradation occurred over time. Furthermore, the zoomed-in views taken at the beginning and end of the switching tests confirm that the response times remain unchanged. Extended stability testing over 10^5^ cycles (Figure S8) confirms that the rise and fall times consistently remain below the 10 μs sampling resolution limit (imposed by data acquisition constraints), demonstrating highly stable switching performance without signal degradation.

**Figure 9:**
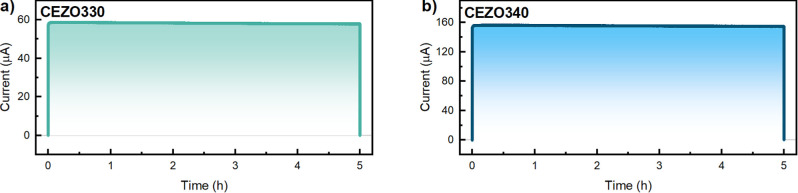
Stability of photocurrent generation via the photovoltaic effect in (a) CEZO330 and (b) CEZO340 samples. The measurements were conducted under 625 nm diode illumination with a sampling frequency of 1 Hz.

**Figure 10:**
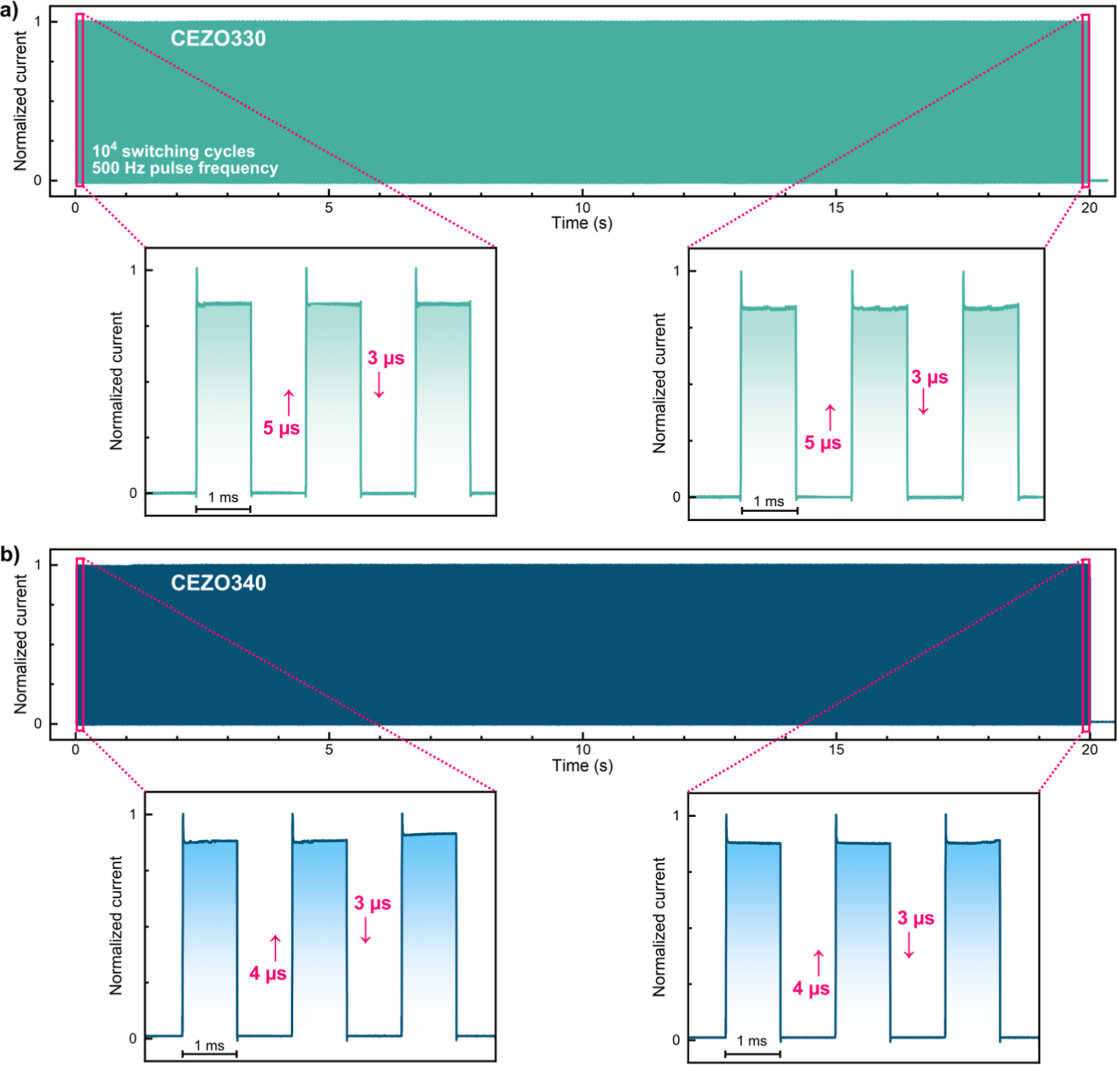
Cyclic switching tests of (a) CEZO330 and (b) CEZO340 samples performed under 650 nm illumination with an intensity of 162 mW/cm^2^. Each test consisted of 10^4^ cycles using 500 Hz light pulses. The zoomed-in graphs present the current–time responses recorded at the beginning and at the end of each test.

The ultrashort response times qualify the investigated structures as ultrafast photodetectors. Table 1 compares the response times, responsivity, and detectivity of various self-powered PDs based on ZnO/Si heterojunctions reported in the literature. The detectivity, D*, of the investigated samples was calculated according to^64^

12 $$D^{*}=\frac{R}{\sqrt{2q\cdot \frac{I_{dark}}{A}}}$$

where *A* is the active illuminated area of the device and *I*_dark_ is the current measured at 0 V under dark conditions (cf. Figure S6). The devices presented in this work belong to the fastest ZnO-/Si-based photodetectors with relatively high photocurrent generation, highlighting the potential of MBE-grown ZnCdO structures for the development of next-generation ultrafast PDs.

**Table 1:** Comparison of Response Times, Responsivity, and Detectivity of Self-Powered PDs Based Solely on ZnO/Si Heterojunctions, Incorporating Either Undoped or Doped ZnO^a^

		response time				
structure	wavelength (nm)	τ_r_	τ_f_	responsivity (mA/W)	detectivity (Jones)	reference
Ag/ZnO/Si/Ag	365	1 s	0.9 s	5	-	^54^
Ag/ZnO:Y NWs/Si/Al	374	420 ms	160 ms	up to 200	1.48 × 10^12^	^65^
ITO/ZnMnO/Si/ITO	900	3.4 ms	4.1 ms	100	∼4 × 10^13^	^66^
ITO/ZnO/Si/In	405	130 μs	120 μs	up to 600	7.7 × 10^12^	^20^
ITO/ZnO NWs/Si/ITO	940	113 μs	200 μs	up to 200	2 × 10^12^	^67^
ITO/ZnO:Ga MW/Si/Ni/Au	370	79 μs	132 μs	200	1.75 × 10^12^	^68^
Al/ZnO NRs/Si/Al	500	64 μs	64 μs	5	1.8 × 10^11^	^30^
ITO/ZnO NWs/Si/Cu	1064	15 μs	21 μs	160	8.78 × 10^11^	^69^
ITO/ZnO/Si/Al	850	10 μs	31 μs	6	3.2 × 10^11^	^29^
Au/ZnCdO:Eu/Si/Al	405	8 μs	3 μs	70	1.01 × 10^11^	this work (CEZO330)
	650	5 μs	3 μs	135	1.93 × 10^11^	
	405	9 μs	2 μs	185	5.52 × 10^11^	this work (CEZO340)
	650	3 μs	3 μs	370	1.09 × 10^12^	

a MW, microwire; NWs, nanowires; NRs, nanorods. The response times presented correspond to the best performance achieved under illumination intensities of 251 mW/cm^2^ at 405 nm and 540 mW/cm^2^ at 650 nm.

## Conclusions

This work reports Eu-doped ZnCdO random alloys grown on Si by molecular beam epitaxy. Presence of Eu^3+^ ions in the structure was evidenced by the PL spectra. Eu and Cd, as foreign elements for the ZnO lattice, can cause its distortion. As a result, defected crystal lattice was revealed by means of cathodoluminescence and Raman spectra.

*I*–*V* curves show that introduction of Cd into ZnO enables ohmic-like behavior of the Au contact. Consequently, structures with the highest Cd concentration exhibit a rectifying ratio at the levels of 110 and 1700 measured at ±4 V. Moreover, these samples display high responsivity in a broad spectral range 370–1150 nm, reaching over 400 mA/W for 700 nm without any external bias, making the presented PDs attractive for energy-saving optoelectronics. Pyro-phototronic effect led to ultrafast reaction times of the PDs. The fastest detection was recorded for the sample with the highest Cd concentration illuminated by 650 nm light, 3 μs for both rise and fall times. These results belong to the shortest detection times presented in the literature for self-powered PDs based solely on the ZnO/Si junction. Furthermore, the ultrafast detection was stable over a wide range of light intensities. The samples can endure at least 10^5^ on/off cycles at 500 Hz without any degradation of signal or response time. All of the described features allow utilization of the proposed structures to detect fast-changing signals under various light conditions (when it comes to both wavelength and intensity) without the need of using any power source.

This study presents a novel approach to revealing the interaction between Cd and Eu doping in ZnO layers and the pyro-phototronic effect, offering valuable insights for improving the response of ZnO-based self-powered photodetectors in fast photosensing applications.

## Supplementary Material



## Data Availability

Data will be made available on request.

## Notes

SIMS data measured for the CEZO340 sample; SIMS data for the ZnO:Eu reference sample; EDX measurements; high-resolution XRD reciprocal space maps; frequency dependence of RC time constant; 0 V dark current measurements; unnormalized data used to determine the response time; and 10^5^ cycle endurance test (PDF)
