# Supplementary material for: ZnCdO:Eu Epitaxially Grown Alloys for Self-Powered Ultrafast Broadband Photodetection
Source: ACS Appl Mater Interfaces. 2026 Mar 25;18(13):19339–52. doi: 10.1021/acsami.6c01143 (PMC13298809; doi:10.1021/acsami.6c01143)
Supplement: Supplementary file 1 [file am6c01143_si_001.pdf]

Supporting Information for ZnCdO:Eu Epitaxially Grown Alloys for Self-Powered Ultrafast Broadband Photodetection

Igor Perlikowski[1], Eunika Zielony[1], Aleksandra Wierzbicka[2], Adrian Kaim[1], Anastasiia Lysak[2], Rafał Jakieła[2], Yaroslav Zhydachevskyy[2], Ewa Przeździecka[2]*

[1] Department of Experimental Physics, Wrocław University of Science and Technology, Wybrzeże Wyspiańskiego 27, 50-370 Wrocław, Poland

[2] Institute of Physics, Polish Academy of Sciences, Al. Lotników 32/46, 02-668 Warsaw, Poland

* *Corresponding author: igor.perlikowski@pwr.edu.pl*

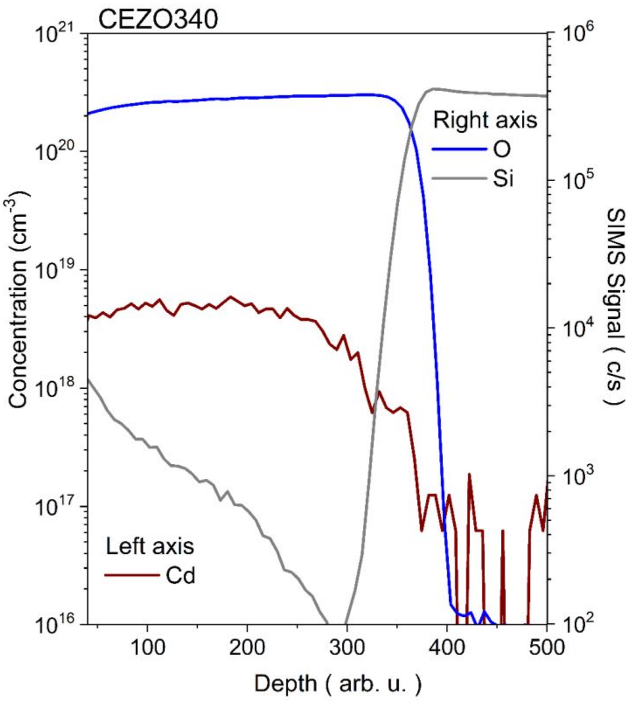

Figure S1. SIMS profile of CEZO340 sample with calculated Cd concentration.

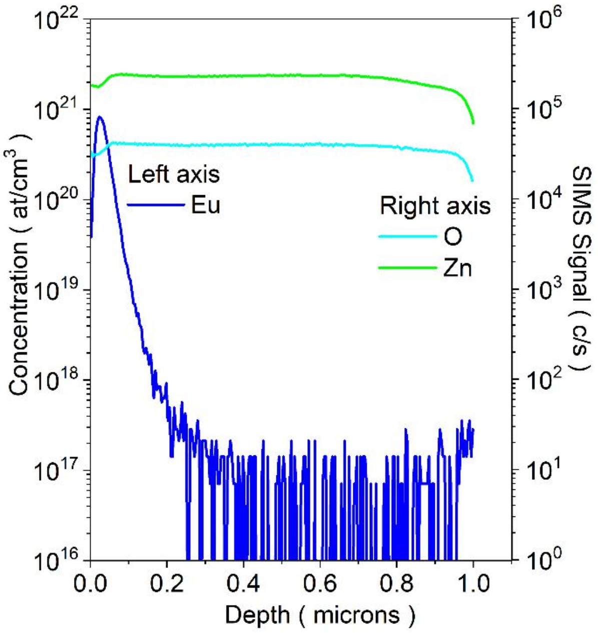

Figure S2. SIMS measurement of a ZnO reference sample doped with europium by ion implantation, showing the Eu detection limit in ZnO at the level of 10^{17} cm^{-3}.

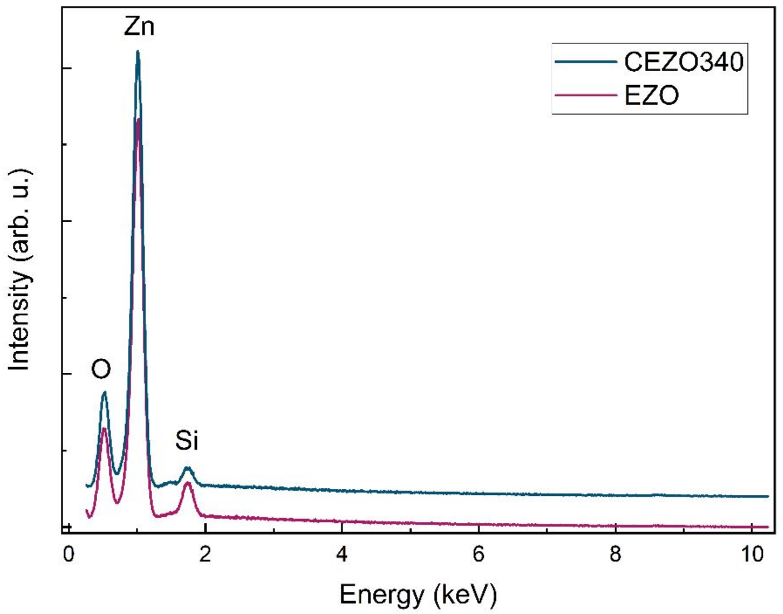

Figure S3. EDX spectra of EZO and CEZO340 samples.

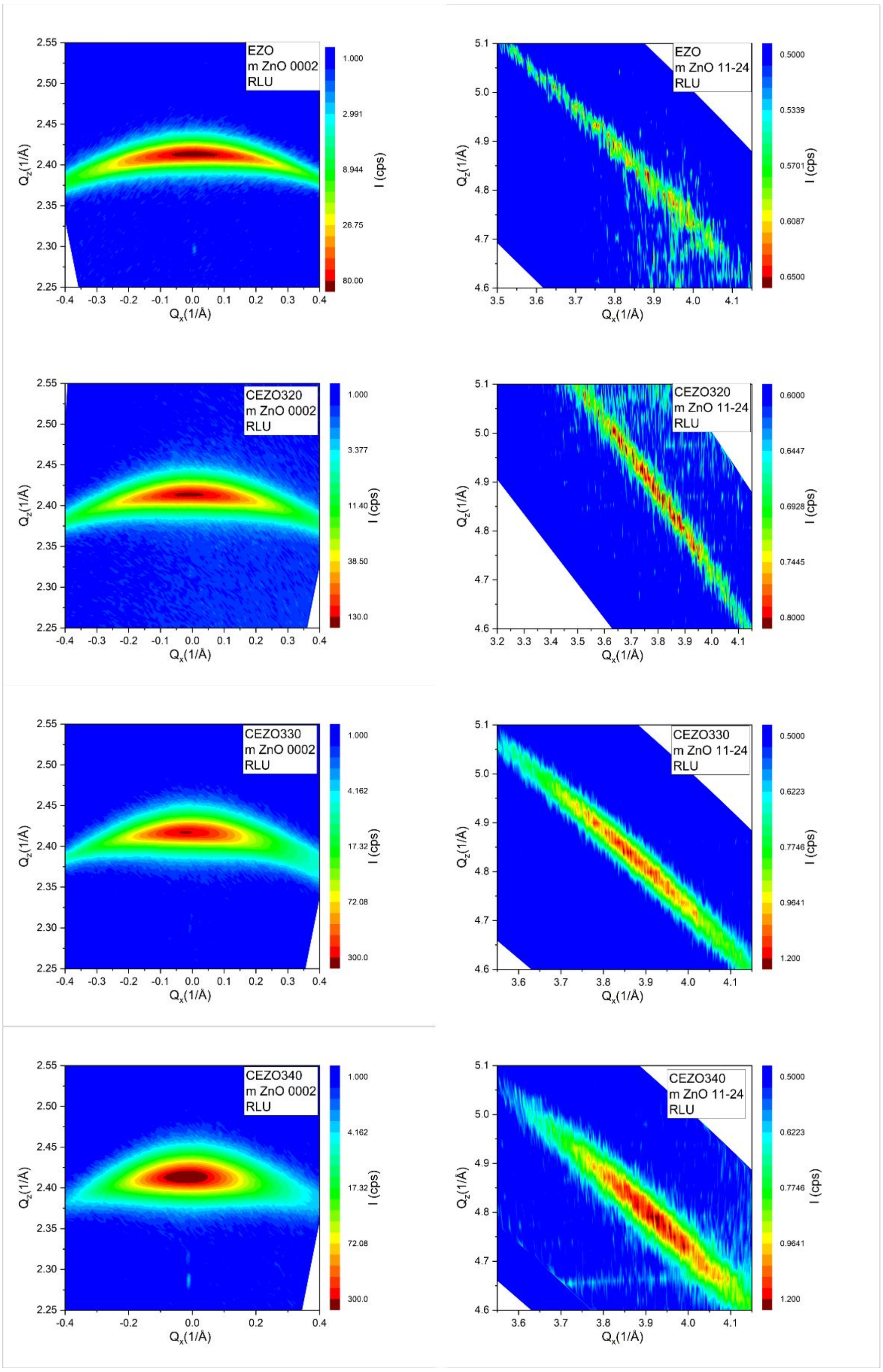

Figure S4. High-resolution XRD reciprocal space maps used for strain calculations.

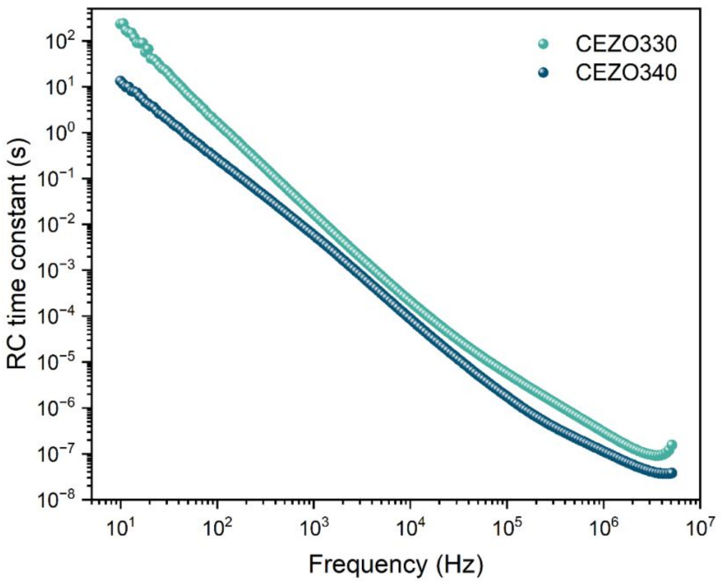

Figure S5. Variation of the RC time constant as a function of the frequency of the test signal with an amplitude of 100 mV.

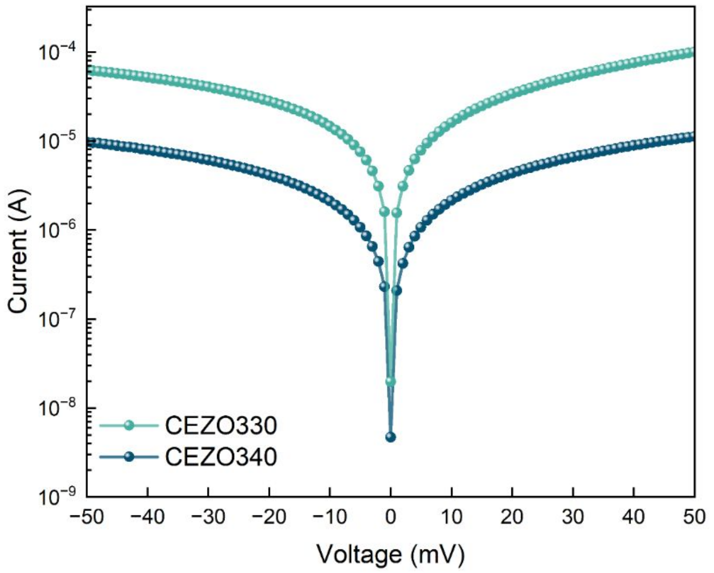

Figure S6. Dark current-voltage measurements performed to extract current values at 0V.

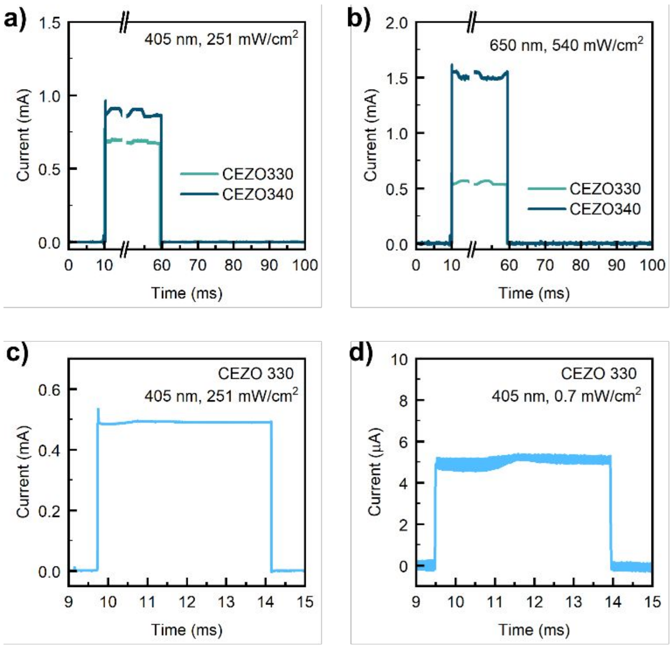

Figure S7. Raw transient photocurrent curves used to extract rise and fall times under (a) 405 nm and (b) 650 nm illumination, and under 405 nm excitation at (c) high and (d) low irradiance levels.

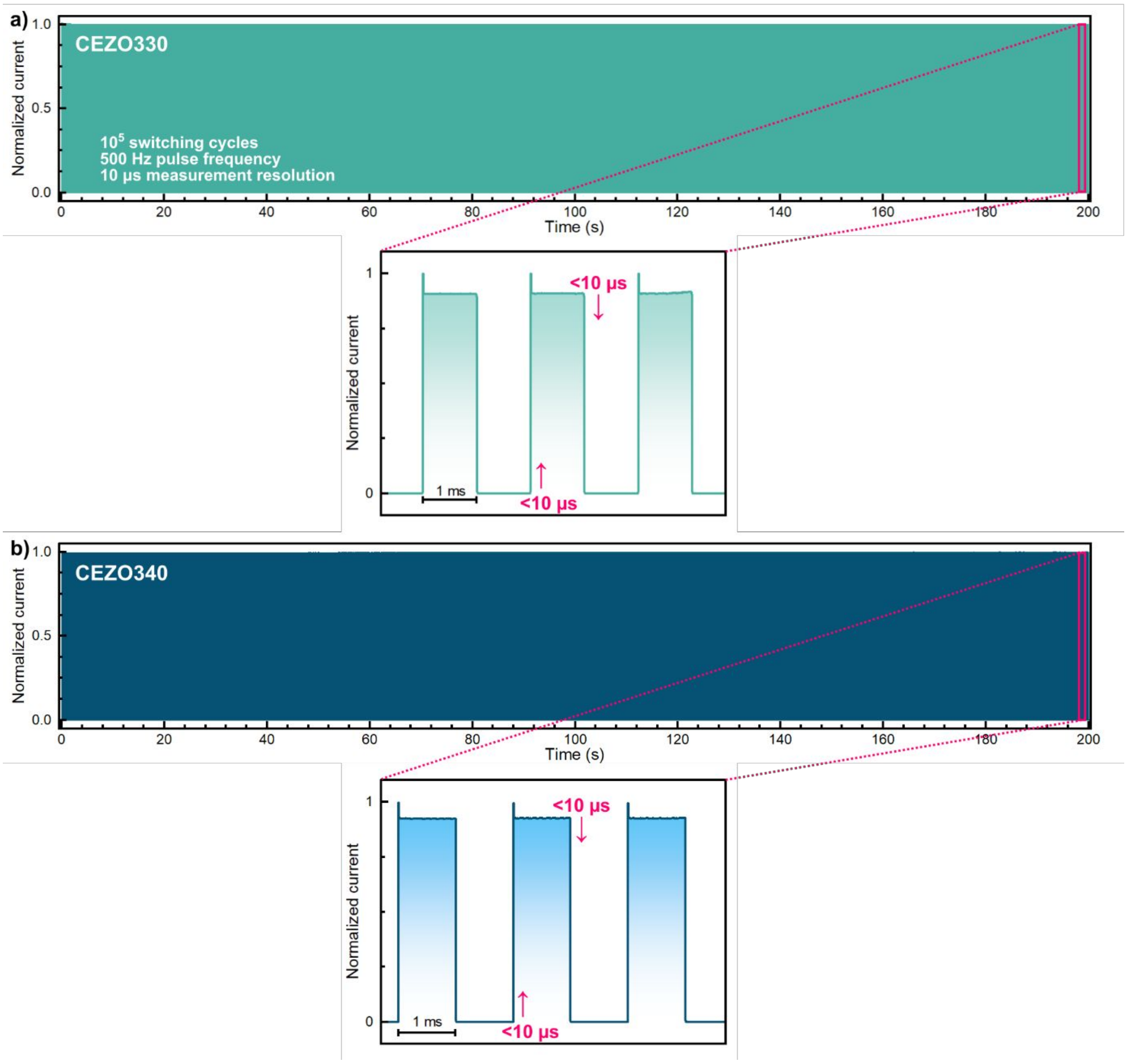

Figure S8. Cyclic switching tests of (a) CEZO330 and (b) CEZO340 samples performed under 650 nm illumination with an intensity of 540 mW/cm^2. Each test consisted of 10^5 cycles using 500 Hz light pulses with 10 µs measurement resolution. The zoomed-in graphs present the current–time responses recorded at the at the end of each test, confirming that the rise and fall times remains under 10 µs after almost 10^5 cycles.
